# A Saving Salt

**Published:** 1875-10

**Authors:** 


### A SAVING SALT.

The theory exists among leading physiologists that the singular substance called phosphorus is the chief agent for conferring what is known as "brain-power" upon the individual, and that the absence of phosphorus is a symptom of lessened mental capacity. It is said that dissection shows two classes nearly or quite devoid of this peculiar salt—the insane and the greatly debilitated.

A healthy human brain is known to contain an appreciable quantity of this substance. If you place the brain of a person who has met his death from some accident which has proved instantaneously fatal, in a retort after weighing it, and condense the escaping vapor, you will discover that four-fifths of that structure by weight is nothing but pure water. If you then follow up your analysis, you will discover that one-fortieth part of all that remains is composed of three salts of phosphorus—lime, potash, and magnesia. If you submit the brain of the lower animals to the same tests, you will fail to find phosphorus until you reach those of the ape class, where you will find it in minute quantities. You will, however, discover it in the bones of all the animal creation, and in those of fishes.

It is evident that man has been supplied with this salt for some purpose. The fact that scarcely a trace of it remains in many diseases of body and mind, indicates clearly that its existence in proper quantity is a condition of health, mental and physical. If this is true, as it seems to be, then it is fair to assume that by replacing the exhausted phosphorus we do much to assist recovery.

If we ask ourselves how this lost phosphorus is to be replaced, we shall have to answer that it may be slowly recovered by the use of phosphatic foods, such as the whole cereals. But we shall have to admit that this process will be inadequate to the building up of systems greatly wasted, of brains badly dismantled. We must seek other and more speedy methods. We can not introduce phosphorus in its pure form, as the potent salt is an irritant in its natural condition, and can not safely be taken into the stomach. In the early period of its use as a medicine, doctors administered it as a phosphate, with but small results. Phosphate of lime acquired a limited reputation as a remedy in lung-diseases, but the results of its use were not largely satisfactory. It was believed that the potency of phosphorus as a remedial agent, must be secured by its administration in some other form. At length, in 1855, Dr. Churchill, of Paris, introduced the "hypophosphites," a soluble form of phosphorus, combined with any appropriate medicinal base. Phosphorus in this form is unoxydized; its oxydation, therefore, takes place in the blood, as it should. It is instantly absorbed and assimilated, and its power is therefore at once felt. Dr. Churchill discovered that the hypophosphites were capable of arresting the progress of many diseases, among them consumption, bronchitis, indigestion, impoverishment of the blood, liver-complaint, rickets and other ailments of young children, and a number of diseases from which woman alone suffers, and presented his discovery to the world, in a paper which he read to the Imperial Academy of Medicine, in Paris, in the summer of 1857. Since that period these remedies, prepared according to Dr. Churchill's formula, have been used with very gratifying results all over Europe.

An expert chemist, Mr. J. Winchester, of No. 36 John Street, New York, shortly after began the manufacture of these phosphorus solutions for the use of the medical faculty in America. In his laboratory all the delicate manipulations required for the production of these active remedies are skillfully performed. Experience and a great mass of testimony has shown that the medicines prepared by him may be employed with safety in the various diseases over which they have manifested specific power, without the intervention of a medical man. Thousands upon thousands of families keep them constantly at hand, for use in emergencies. Of the multitude of diseased states in which Winchester's Hypophosphites may be employed with benefit, we have not space to speak. Mr. Winchester will mail descriptive pamphlets to all who would investigate this important subject. We would merely add that our experience gives us great confidence in the value of this Saving Salt—phosphorus—as prepared for use as a medicine by this gentleman.

